# Effect of air removal with extracorporeal balloon inflation on incidence of asymptomatic cerebral embolism during cryoballoon ablation of atrial fibrillation: A prospective randomized study

**DOI:** 10.1016/j.ijcha.2022.101020

**Published:** 2022-04-07

**Authors:** Masaaki Yokoyama, Michifumi Tokuda, Kenichi Tokutake, Hidenori Sato, Hirotsuna Oseto, Kenichi Yokoyama, Mika Kato, Ryohsuke Narui, Shin-ichi Tanigawa, Seigo Yamashita, Michihiro Yoshimura, Teiichi Yamane

**Affiliations:** Department of Cardiology, The Jikei University School of Medicine, Japan

**Keywords:** Atrial fibrillation, Ablation, Cryoballoon, Pulmonary vein isolation, Cerebral embolism, Complication

## Abstract

**Background:**

It was previously reported, based on a retrospective study, that preliminary removal of air bubbles in heparinized saline water with extracorporeal balloon inflation reduced the incidence of asymptomatic cerebral embolism (ACE). The present study aims to compare the incidence of ACE between a conventional and pre-inflation method during cryoballoon ablation in a prospective randomized controlled study.

**Methods:**

A total of 98 atrial fibrillation patients were enrolled and randomized into conventional and pre-inflation groups. Patients in the pre-inflation group received balloon massaging with preliminary extracorporeal balloon inflation in saline water before the cryoballoon was inserted into the body.

**Results:**

The baseline characteristics were similar between the two groups. Post-procedural 3-Tesla MRI revealed CE in 27.6% of patients. Symptomatic CE only occurred in two patients in the pre-inflation group. One patient had transient dysarthria and mild muscle weakness in one hand; the other patient complained of transient left upper limb weakness, left lower limb paresthesia and dysarthria. The incidence of ACE detected by cerebral MRI did not differ between the two groups to a statistically significant extent (conventional vs. pre-inflation; 22.9% vs. 29.2%; P = 0.49). In the multivariable analysis, eGFR was independently associated with the presence of ACE (odds ratio 0.95; 95% confidence interval 0.907–0.995; P = 0.03).

**Conclusion:**

In this prospective randomized study, the preliminary removal of air bubbles in heparinized saline water with extracorporeal balloon inflation had no impact on the incidence of ACE.

## 1 Introduction

Cryoballoons have proven to be effective for pulmonary vein isolation (PVI) in patients with atrial fibrillation (AF) [1], [2]. Several recent randomized trials have shown the noninferiority of cryoballoon ablation to radiofrequency ablation with respect to the treatment efficacy in patients with drug-refractory paroxysmal AF [1], [2].

Several complications related to catheter ablation are recognized, including cardiac tamponade, phrenic nerve injury, PV stenosis, and esophageal injury [1], [3]. Symptomatic cerebral embolism occurred during cryoballoon ablation of AF in 0–0.5% of patients [1], [2], [3], [4], [5], [6], [7], [8], [9]. However, procedural asymptomatic cerebral embolism (ACE) was detected by cerebral magnetic resonance imaging (MRI) in 4–27% of patients after cryoballoon ablation of AF. A previous study reported that the incidence of ACE was not markedly different between patients who received radiofrequency ablation of AF and those who underwent cryoballoon ablation of AF [6]. Both microparticles (clots and char) and microbubbles (air and gas) have been considered as sources of cerebral embolisms during catheter ablation procedures [10]. Air bubble intrusion during the catheter ablation process is mainly a procedure-related complication. In particular, before the insertion of a cryoballoon catheter into the femoral vein sheath, massaging of the cryoballoon in order to remove air bubbles may be crucial to reducing the risk of ACE.

Since the cryoballoon is tightly folded to pass through the cryoballoon sheath, air bubbles may be hidden deep in the folded balloon surface. We detected multiple air bubbles, despite careful conventional balloon massaging having been performed in all cases. Balloon massaging in saline water with balloon inflation can remove air bubbles completely before insertion into the body. In a previous survey by propensity score matching, we reported that air removal with pre-inflation resulted in a lower incidence of ACE in comparison to the conventional air removal method, whereas another study reported a negative result [11], [12]. The aim of this study was to investigate the impact of the pre-inflation air removal method on the incidence of ACE during cryoballoon ablation of atrial fibrillation in a prospective randomized trial.

## 2 Methods

### 2.1 Study population

This was a prospective, single-center, single blinded, randomized study. A total of 98 consecutive AF patients who were scheduled to undergo initial PVI by cryoballoon were included in the present study. The study was approved by the Human Research Committee of The Jikei University School of Medicine (29-091(8707)). All patients were recruited at a single center (The Jikei University Hospital). All patients gave their written informed consent. Patients were electronically randomized at a 1:1 ratio to the conventional air removal method or the pre-inflation air removal method. The patients were randomly assigned to two groups with simple random sampling using a computerized random number generator. The conventional and pre-inflation air removal methods have been described previously [11]. Before the cryoballoon was inserted into the body, patients who were assigned to conventional group underwent conventional massaging while the balloon remained folded for air removal in heparinized (1000U of heparin in 1000 mL of 0.9% NaCL) saline water. In patients assigned to the pre-inflation group, pre-inflation massaging in heparinized saline water with extracorporeal cryoballoon inflation was performed. Antiarrhythmic drugs were discontinued at least 5 half-lives before ablation. Transesophageal echocardiography and/or multi detector computed tomography (MDCT) was performed before the procedure to rule out left atrial thrombus.

### 2.2 Periprocedural anticoagulation

Anticoagulants were administered at least 3 weeks before the procedure and were continued during the procedure. Intravenous unfractionated heparin was administered immediately after the insertion of the sheath and was maintained throughout the ablation procedure to maintain an activated clotting time (ACT) of ≥300 s. Ablation was delayed until ACT ≥ 300 s had been reached. The ACT was monitored every 20 min. If the ACT fell to <300 s during the procedure, an additional bolus of heparin (1000–2000 U) was administered.

### 2.3 Cryoballoon ablation of AF

Cryoballoon ablation of AF was performed as previously described [11], [13]. An esophageal temperature probe was inserted throughout the ablation procedure to avoid esophageal injury. A single transseptal puncture was performed using a radiofrequency needle (Baylis Medical, Montreal, Canada). An 8-Fr sheath (SL0; St. Jude Medical) and cryoballoon sheath (Flexcath Advance®; Medtronic, Minneapolis, MN, USA) were inserted to the left atrium via a single puncture site. A 15–25-mm circular mapping catheter was used to map all pulmonary veins (PVs) before and after the cryoballoon to confirm electrical isolation. PVI was performed using a 180 s single balloon technique with a second-generation cryoballoon. A 28-mm cryoballoon catheter (Arctic Front Advance®; Medtronic) was used in all patients. A spiral mapping catheter (Achieve, Medtronic) was used to advance the cryoballoon and to map the PV potentials. When inserting a cryoballoon or other catheter into the sheath, we manually evacuated the air in the sheath using a 20-ml syringe and slowly injected heparinized saline water. Complete sealing at the antral aspect of the PV was confirmed by the injection of contrast medium. This was followed by a freeze cycle of 180 s. To avoid phrenic nerve injury, the diaphragmatic compound motor action potentials were monitored during phrenic nerve pacing while all the cryoballoon applications (180 s per application) per vein, additional touch-up ablation was performed with a radiofrequency catheter.

### 2.4 Postprocedural cerebral MRI

In all cases, cerebral MRI was performed using a 3-Tesla scanner on the one day after the ablation procedure. The imaging protocol for all images consisted of a T2-weighted axial fluid-attenuated inversion recovery (FLAIR) sequence and a diffusion-weighted MRI (DWI) sequence. For each DWI sequence, the apparent diffusion coefficient (ADC) map was obtained to rule out any shine through artifact. The presence/absence of acute embolism was evaluated by independent, blinded neuro-radiologists. MRI findings were defined as follows: cerebral embolism = DWI positive + ADC reduced

A systematic clinical neurological examination was performed by a certified neurologist or certified physician who had sufficient experience regarding neurological examinations and who were blinded to this study.

### 2.5 Statistical analyses

Continuous data are expressed as the mean ± SD for normally distributed variables or as the median (25th–75th percentile) for non-normally distributed variables, and were compared using Student’s *t*-test or the Mann-Whitney *U* test, respectively. Categorical variables, expressed as numbers or percentages, were compared using a χ^2^ test, unless the expected value in any cells was <5, in which case Fisher’s exact test was used. P-values of <0.05 were considered to indicate statistical significance.

Multivariable analysis using a multiple regression were performed to assess the predictors of preprocedural ACE. That was used to calculate odds ratios and 95% confidence intervals after controlling simultaneously for potentially confounding factors of age, eGFR, BNP and AF history. All statistical analyses were performed using the SPSS software program (version 25.0.0; SPSS, Chicago, IL, USA).

## 3 Results

### 3.1 Patient characteristics and procedural details

Conventional and pre-inflation air removal methods were performed in 48 patients and 50 patients, respectively. The baseline patient characteristics were similar between the two groups (Table 1). The total procedure time and freezing time of the conventional and pre-inflation groups were similar (131 ± 33 min vs. 134 ± 30 min; P = 0.61 and 13.6 ± 3.5 min vs. 14.3 ± 3.2 min; P = 0.29, respectively) (Table 1). Electrical defibrillation of AF was similarly performed (P = 0.32), touch-up ablation was similarly required in both groups (P = 0.11). There was no significant difference between the two groups in the initial ACT value or in the rate of subsequent ACT values of <300 s (232 ± 35 s vs. 246 ± 46 s; P = 0.09 and 15(31%) vs. 16(32%); P = 0.94).

**Table 1:** Patient characteristics and procedural details.

	Conventional N = 48	Pre-inflation N = 50	P value
Patient characteristics			
Sex (male)	36(75%)	34(68%)	0.44
Age (years)	60.9 ± 9.6	62.5 ± 11.9	0.48
Body mass index (kg/m^2^)	24.0 ± 2.7	23.7 ± 2.9	0.67
LA diameter (mm)	37.0 ± 5.4	35.8 ± 4.9	0.26
LV ejection fraction (%)	63.6 ± 7.1	65.6 ± 5.7	0.14
eGFR (ml/min/1.73 m^2^)	72.8 ± 15.6	74.8 ± 12.3	0.48
BNP (pg/ml)	68.1 ± 81.5	61.3 ± 64.8	0.65
History of AF (years)	2.0(0.8–4.0)	2.0(1.0–5.0)	0.52
Hypertension	24(50%)	22(44%)	0.55
Diabetes mellitus	3(6%)	3(6%)	1.00
Heart failure	4(8%)	3(6%)	0.71
Old cerebral infarction	1(2%)	4(8%)	0.36
CHADS_2_ score	0.8 ± 0.9	0.9 ± 1.1	0.67
Use of DOAC	47(98%)	49(98%)	1.00
Procedural details			
Electrical defibrillation for AF	19(40%)	15(30%)	0.32
Total procedure time (min)	131 ± 33	134 ± 30	0.61
Total freezing time (min)	13.6 ± 3.5	14.3 ± 3.2	0.29
Major complication	7(15%)	4(8%)	0.35
Right phrenic nerve palsy	4	3	0.71
Symptomatic cerebral embolism	0	2	0.5
AV shunt, hematoma	2	0	0.24
Pneumothorax	1	0	0.49
PV stenosis	1	0	0.49
Jaw thrust	22(46%)	22(44%)	0.86
Insertion of oropharyngeal airway	16(33%)	9(18%)	0.08
Phrenic nerve palsy	3(5.9%)	4(7.0%)	1.00
RF ablation in LA (non-PV)	2(4.2%)	3(6.0%)	1.00
Touch-up ablation	7(14.6%)	14(28%)	0.11
Catheter (other than CB) insertion to LA via FlexCath Advance	3(6.3%)	1(2.0%)	0.36
Minimum ACT value < 300 sec	15(31%)	16(32%)	0.94
Initial ACT value (sec)	232 ± 35	246 ± 46	0.09

The data are presented as the mean ± SD, n (%) or median (25th–75th percentile). LA, left atrium; LV, left ventricle; eGFR, estimated glomerular filtration rate; BNP, B-type natriuretic peptide; AF, atrial fibrillation; DOAC, direct acting anti-coagulants. AF, atrial fibrillation; AV, arteriovenous; PV, pulmonary vein; RF, radiofrequency; CB, cryoballoon; ACT, activated clotting time.

### 3.2 Cerebral embolism

**Fig. 1:**
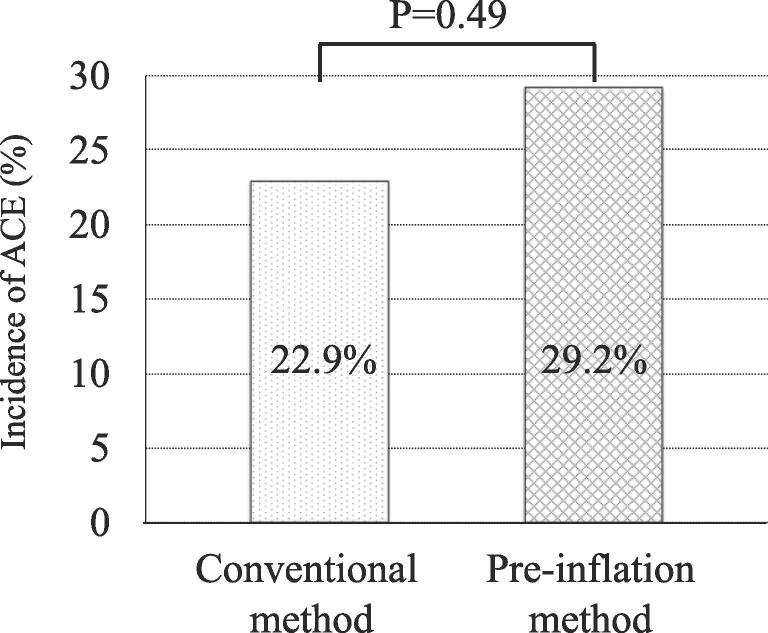
Incidence of asymptomatic cerebral embolism. The incidence of asymptomatic cerebral embolism in the pre-inflation and conventional groups did not differ to a statistically significant extent. ACE, asymptomatic cerebral embolism.

As an adverse event, symptomatic cerebral embolism was observed in 2 cases in the pre-inflation group. One patient had dysarthria and mild muscle weakness in one hand. The other patient complained of left upper limb weakness, left lower limb paresthesia and dysarthria. Their symptoms improved within one month and follow-up MRI showed no evidence of cerebral hemorrhage or an exacerbation of cerebral embolism. The incidence of ACE (except for symptomatic CE) in the pre-inflation and conventional groups did not differ to a statistically significant extent (14(29.2%) vs. 11(22.9%); P = 0.49) (Fig. 1). The incidence of CE (including asymptomatic CE and symptomatic CE) in the pre-inflation and conventional groups did not differ to a statistically significant extent (16(32.0%) vs. 11(22.9%); P = 0.31).

Table 2 shows the patient characteristics in patients with and without ACE. The patients with ACE were older, lower in eGFR, higher in BNP and had a longer history of AF in comparison to those without ACE. Table 2 shows also the procedural details between the patients with and without ACE. There were no significant differences in the procedural factors of both groups. In a multivariable analysis, the eGFR was the sole factor that was independently associated with the presence of ACE (odds ratio 0.95; 95% confidence interval 0.907–0.995; P = 0.03) (Table 3).

**Table 2:** Baseline characteristics and procedural details of patients with and without ACE.

	ACE (−) N = 71	ACE (+) N = 25	P value
Baseline characteristics			
Sex (male)	50(70%)	20(80%)	0.35
Age (years)	59.8 ± 10.8	66.6 ± 9.6	0.01
Body mass index (kg/m^2^)	24.0 ± 2.9	23.5 ± 2.3	0.46
LA diameter (mm)	36.2 ± 5.4	37.1 ± 4.7	0.46
LV ejection fraction (%)	64.3 ± 6.5	64.9 ± 6.3	0.69
eGFR (ml/min/1.73 m^2^)	76.7 ± 14.3	65.6 ± 11.4	<0.01
BNP (pg/ml)	53.3 ± 57.6	98.7 ± 101.5	0.04
History of AF (years)	1.0(0.8–3.0)	3.0(1.0–5.0)	0.03
Hypertension	31(44%)	13(52%)	0.47
Diabetes mellitus	5(7.0%)	1(4.0%)	1.00
Heart Failure	5(7.0%)	2(8.0%)	1.00
Old cerebral infarction	4(5.6%)	0(0%)	0.57
CHADS_2_ Score	0.8 ± 1.0	0.9 ± 1.0	0.54
Use of DOAC	70(99%)	24(96%)	0.46
Procedural details			
Balloon massaging with extracorporeal balloon inflation	34(48%)	14(56%)	0.49
Electrical defibrillation during procedure	21(30%)	11(44%)	0.19
Touch-up ablation (PVs)	0.3 ± 0.6	0.2 ± 0.4	0.20
Total procedure time (min)	131 ± 32	134 ± 29	0.71
Total freezing time (min)	13.8 ± 3.2	14.2 ± 3.4	0.51
Jaw thrust	31(44%)	12(48%)	0.71
Insertion of oropharyngeal airway	17(24%)	7(28%)	0.69
Phrenic nerve palsy	4(5.6%)	2(8.0%)	0.65
RF ablation in LA (non-PV)	3(4.2%)	2(8.0%)	0.60
Touch-up ablation	17(24%)	4(16%)	0.58
Catheter (other than CB) insertion to LA via FlexCath Advance	4(5.6%)	0(0%)	0.57
Minimum ACT value < 300 sec	24(34%)	7(28%)	0.59
Initial ACT value (sec)	238 ± 43	242 ± 36	0.68

The data are presented as the mean ± SD, n (%) or median (25th–75th percentile).

ACE, asymptomatic cerebral embolism; LA, left atrium; LV, left ventricle; eGFR, estimated glomerular filtration rate; BNP, B-type natriuretic peptide; AF, atrial fibrillation; DOAC, direct acting anti-coagulants; AV, arteriovenous; PV, pulmonary vein; RF, radiofrequency; CB, cryoballoon; ACT, activated clotting time.

**Table 3:** Multivariate analysis of predictors of asymptomatic cerebral embolism.

	Odds ratio	95 %CI	P value
Age	1.046	0.985–1.111	0.14
eGFR	0.95	0.907–0.995	0.03
BNP	1.006	0.999–1.013	0.08
AF history	1.035	0.966–1.110	0.32

CI, confidence interval; eGFR, estimated glomerular filtration rate; BNP, B-type natriuretic peptide; AF, atrial fibrillation.

## 4 Discussion

### 4.1 Brief summary

This is the first study to evaluate the benefits of the pre-inflation air removal method on the incidence of ACE during cryoballoon ablation of AF in a prospective randomized trial. The pre-inflation method did not reduce the incidence of ACE during cryoballoon ablation of AF.

### 4.2 Air removal with extracorporeal balloon inflation

In a previous study reported from our institute, extracorporeal air removal reduced the incidence of ACE during cryoballoon ablation [11]. This method has been widely practiced in some areas and is recommended to be performed with a novel cryoballoon system (POLARx, Boston Scientific, St. Paul, MN, USA). However, from this randomized study, the merit of the pre-inflation cannot be proved. Another previous single-center observational study did not report the superiority of the pre-inflation method [12]. On the other hands, an *in vitro* study reported that an air removal with extracorporeal balloon inflation in saline before insertion reduced the number of small and large air bubbles in comparison to temporary balloon massaging [14]. Since our previous study was a retrospective non-randomized study, it was possible that it involved some bias.

Air embolism is one of the causes of cerebral embolism during catheter ablation. Thrombi, gas bubbles, and particulate debris (coagulum) have been found to be introduced or generated in the left atrium during catheter ablation of AF, and cerebral lesions can be experimentally generated by solid small-sized particles or gaseous microbubbles [10]. Although the pre-inflation method can reduce the risk of air emboli at the time of initial cryoballoon inflation, it cannot prevent other causes of CE. Symptomatic CE occurred in two patients in the pre-inflation group. It was also possible that the pre-inflation caused poor folding of the cryoballoon, which increased bubble intrusion from the sheath during balloon insertion, offsetting the benefits of pre-inflation. It was previously reported that a massive intrusion of large air bubbles was observed when an inserter was used for circular mapping catheter insertion *in vitro* [14]. In addition, our previous study was performed with 1.5 Tesla MRI. Since this study was performed with a 3 Tesla MRI, it is possible that a smaller embolus was also projected.

### 4.3 Predictors of cerebral embolism during cryoballoon ablation

It has been pointed out that ACE caused by air contamination is affected by insertion of catheters of different sizes from the sheath, radiofrequency catheter ablation in the left atrium, and negative pressure in the thoracic cavity [7], [14], [15]. The extended and complex catheter shape allows more air to be introduced across the valve of the sheath in comparison to the smooth bullet shape of a typical radiofrequency ablation catheter. However, in this study, touch-up radiofrequency ablation, catheter insertion to the LA via cryoballoon sheath, the frequency of jaw thrust, and airway insertion were not associated with the incidence of ACE.

For more accurate confirmation of PVI, we use a Lasso catheter in addition to an Achieve catheter. It was reported that after confirming PVI with an Achieve catheter, residual PV potentials were detected by a Lasso catheter in 4.3% of all PVs, 20% of which are located in the RIPV [16]. However, serial Lasso catheter exchange in LA may be associated with the occurrence of air embolism. The signal of a microembolism detected by transcranial Doppler monitoring increased around the time of Lasso mapping [17]. Although the results are different from those obtained by Yokoyama et al. [16], a previous study showed no loss of accuracy in detecting PVI after cryoballoon ablation when using a 20 mm-Achieve catheter [18]. Cryoballoon procedures can be completed with one cryoballoon and an Achieve catheter. The completion of cryoballoon ablation without Lasso exchange may further reduce the incidence of CE.

Previous reports suggest an association between CE immediately after ablation and age [19], persistent arrhythmia [20], spontaneous echocardiographic contrast on transesophageal echocardiography [6], [21], enlargement of the left atrium [22], and cardioversion during the ablation procedure [23]. The association between the renal function and CE during AF ablation has been unclear. Previous studies reported that silent cerebral infarction was independently associated with the development and progression of chronic kidney disease [24], [25]. Chronic inflammation, oxidative stress, sympathetic nerve overactivity, and thrombogenic factors contribute to the excess risk of cerebrovascular disease in patients with chronic kidney disease by causing vascular injury and endothelial dysfunction.

### 4.4 Limitations

The present study was associated with some limitations. First, it was a single-center study. Second, since preprocedural MRI was not performed in this study, we could not deny the possibility that cerebral embolism occurred immediately before the procedure. However, in previous studies of AF ablation without interruption of anticoagulants, none of the patients had acute CE on preoperative MRI [6], [26]. Therefore, it is likely that most CE lesions observed on postoperative MRI were related to the ablation procedure. It is possible that highly detailed neuropsychological testing may detect subtle neurological changes.

## 5 Conclusion

In a prospective randomized trial, preliminary cryoballoon massaging in heparinized saline water with extracorporeal balloon inflation to remove air bubbles did not reduce the incidence of asymptomatic cerebral embolism during cryoballoon ablation of AF.

Conflict of interest: Michifumi Tokuda received consulting fee from Medtronic. Teiichi Yamane received speaker honoraria from DAIICHI SANKYO COMPANY, Ltd., Japan, Boerringer Ingelheim, Abbott Japan, Medtronic Japan, and Kaneka Corporation and research grants from Boehringer Ingelheim.

## Declaration of Competing Interest

The authors declare that they have no known competing financial interests or personal relationships that could have appeared to influence the work reported in this paper.
